# USP7: Novel Drug Target in Cancer Therapy

**DOI:** 10.3389/fphar.2019.00427

**Published:** 2019-04-30

**Authors:** Zhiru Wang, Wenting Kang, Yinghua You, Jingru Pang, Hongmei Ren, Zhenhe Suo, Hongmin Liu, Yichao Zheng

**Affiliations:** ^1^School of Pharmaceutical Sciences, Zhenghzou University, Zhengzhou, China; ^2^Collaborative Innovation Centre of New Drug Research and Safety Evaluation, Henan Province, and Key Laboratory of Advanced Drug Preparation Technologies, Zhengzhou University, and Key Laboratory of Henan Province for Drug Quality and Evaluation, Ministry of Education of China, Zhengzhou, China; ^3^Pathology, Institute of Clinical Medicine, Faculty of Medicine, University of Oslo, Oslo, Norway

**Keywords:** deubiquitination, USP7, structure, immune, DNA damage

## Abstract

Ubiquitin specific protease 7 (USP7) is one of the deubiquitinating enzymes (DUB) that erases ubiquitin and protects substrate protein from degradation. Full activity of USP7 requires the C-terminal Ub-like domains fold back onto the catalytic domain, allowing the remodeling of the active site to a catalytically competent state by the C-terminal peptide. Until now, numerous proteins have been identified as substrates of USP7, which play a key role in cell cycle, DNA repair, chromatin remodeling, and epigenetic regulation. Aberrant activation or overexpression of USP7 may promote oncogenesis and viral disease, making it a target for therapeutic intervention. Currently, several synthetic small molecules have been identified as inhibitors of USP7, and applied in the treatment of diverse diseases. Hence, USP7 may be a promising therapeutic target for the treatment of cancer.

## Introduction

**FIGURE 1:**
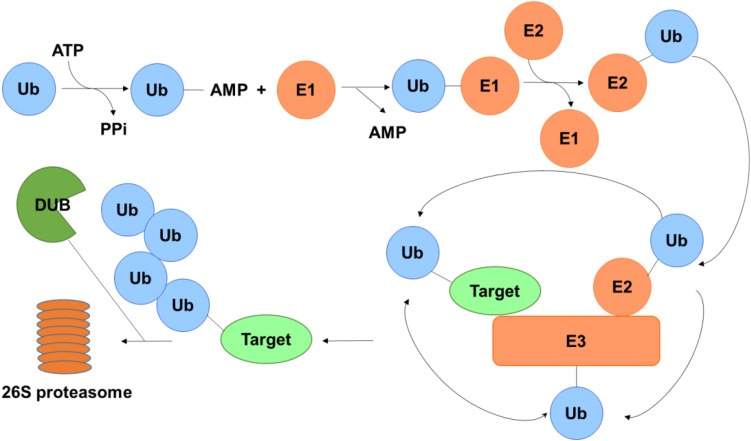
Schematic of the ubiquitin-proteasome system. Ubiquitin is activated by E1 in the presence of ATP and transferred to E2 and then conjugated to a lysine or α-amino group of the substrate protein with the aid of E3. Polyubiquinated targets are recognized and degraded by the 26S proteasome, while the ubiquitin on the substrate can be erased by DUBs to protect it from degradation.

Post-translational modification (PTM) is generally enzymatic modification of proteins following protein biosynthesis. Examples of PTM include methylation, acetylation, phosphorylation, glycosylation, ubiquitination, S-nitrosylation, and so on (Chatterjee and Thakur, 2018). As one of the most studied PTMs, ubiquitination involves in the intracellular proteolytic machinery and regulates numerous physical activities in the cell (Dybas et al., 2018). The process of the addition of ubiquitin to a substrate protein is named ubiquitination, which may contribute to the protein degradation. Ubiquitination of target protein can be catalyzed by a cascade reaction comprising the ubiquitin-activating enzymes (E1), the ubiquitin conjugation enzymes (E2) and the ubiquitin ligases (E3). First, ubiquitin is activated by E1 with the participation of ATP and transferred to E2 through a trans-thiolation reaction, and then conjugated to a lysine or α-amino group of the substrate protein in the presence of E3 (Cheon and Baek, 2006). Eventually, proteins labels with more than four ubiquitin molecules can be recognized and subjected to the 26S proteasome at which they are degraded, generating small polypeptides (Figure 1).

Deubiquitinating enzymes (DUBs) are responsible for the removal of ubiquitin and keeping the stability of the substrate by rescuing them from degradation (Nijman et al., 2005; Clague et al., 2013). Until now, approximately 100 DUBs have been identified and can be classified into five subclasses based on their Ub-protease domains: ubiquitin-specific proteases (USPs), ubiquitin C-terminal hydrolases (UCHs), ovarian tumor proteases (OTUs), Machado-Joseph disease proteases (MJDs) belonging to cysteine-dependent proteases, and JAB1/MPN/Mov34 (JAMMs) belonging to zinc metalloproteases (Zhou et al., 2018). With approximately 50 members, the USPs family is the largest one among all the DUB subfamilies. All these members include conserved domains, i.e., three primary functional domains of Cys, His and Asp/Asn boxes which are in charge of the reorganization of ubiquitin conjugated molecules.

Among the members of USP family, ubiquitin specific protease USP7, also known as herpes-associated ubiquitin-specific protease (HAUSP), is a unique deubiquitinating enzyme which was identified in 1997, and it characterized as a novel member of the ubiquitin-specific protease family to interact with herpes simplex virus type 1 immediate-early protein (Vmw110) of the herpes simplex virus type 1 (HSV-1) regulatory protein (Everett et al., 1997). Later, USP7 was found to interact with other viral proteins such as the Epstein-Barr nuclear antigen 1 (EBNA1) of Epstein-Barr virus (EBV) and the vIRF1 (viral interferon regulatory factor 1) protein of Kaposi’s sarcoma associated herpesvirus (KSHV) (Holowaty et al., 2003), therefore indicating it as a general target of herpes viruses and giving it the name herpes-associated ubiquitin specific protease. Up to now, USP7 is the most widely studied deubiquitinating enzymes, and is considered as an oncogene by promoting tumor growth and negatively affecting the patient immune response to tumors (Everett, 2014; Lu et al., 2016).

## Structure of USP7

**FIGURE 2:**
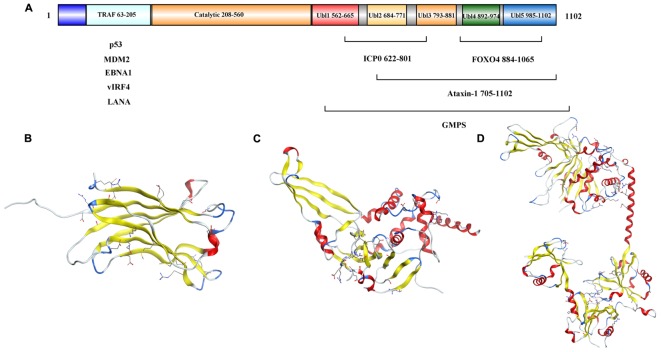
Structure analysis of USP7. **(A)** USP7 primary sequence map. **(B)** Structure of the USP7 N-terminal domain (PDB 2F1W). **(C)** Structure of USP7 catalytic domain and five UBl-domains (PDB 1NB8). **(D)** Structure of the inactive state of USP7 catalytic domain (PDB 5FWI).

The full length USP7 includes 1102 amino acids. There are four domains: an N-terminal poly-glutamine stretch (poly Q), the tumor necrosis factor receptor- associated factors (TRAF) domain (amino acids 62–205), the catalytic domain (amino acids 208–560), and the C-terminal tandem ubiquitin-like (Ubl) domain (amino acids 560–1102) (Kim and Sixma, 2017) (Figure 2A).

As reported, the amino acids 62–205 of USP7 (Figure 2B) bind to EBNA1 (Holowaty et al., 2003), mouse double minute 2 homolog (MDM2) and p53 (Hu et al., 2006; Sheng et al., 2006) through a PA-x-x-S motif (Saridakis et al., 2005), and the TRAF (amino acids 62–205) domain contributes to the nuclear localization of the USP7 (Fernandez-Montalvan et al., 2007). Besides, the USP7 truncation (amino acids 208–1102) performed similar activity as the full length protein (Ma et al., 2010).

Hu et al. (2002) identified a 40 kDa fragment of USP7 as the catalytic domain (amino acids 208–560), which mediates ubiquitin binding and deubiquitination of the substrate. The structure of the catalytic core domain reveals novel three-domain architecture, including Fingers, Palm, and Thumb domains (Figure 2C). This catalytic core domain binds to ubiquitin aldehyde, which reveals a conformational change in the active site (Hu et al., 2002). With the aid of molecular dynamics simulations, it is found that the transition of USP7 from the inactive to the active can only be captured when H294 was neutralized with a deprotonated C223 and charged H464. In the inactive apo state, positively charged H294 stabilizes an electrostatic network with W285, E298, and Y224. However, neutral H294 in the active state cannot make charge interactions, so the electrostatic network is disrupted. That would results in the C223 unfavorable backbone angles improved by helical refolding, thus, the active site is formed (Ozen et al., 2018).

Ubl shares the ubiquitin β-grasp fold, however, it lacks the C-terminal Gly–Gly residues that are required for conjugation to a target and is located outside the boundaries of the catalytic core domain (Faesen et al., 2011). There are five Ubl domains that are detected in the C-terminal and are organized in a 2-1-2 manner as Ubl-12, Ubl-3, and Ubl-45 (Figure 2D) (Zhu et al., 2007). Among them, Ubl-45 is sufficient to reconstitute the USP7 activation *in vitro* and *in vivo*. In the C-terminal, the 19 residues of USP7 (amino acids 1084–1102) are conserved across species (Faesen et al., 2011). Rouge et al. (2016) revealed how the C terminal 19 amino acids of the USP7 contribute to the enhancement of USP7 activity by stabilizing the ubiquitin binding conformation of the catalytic domain. And the individual point mutations at residues I1100 or I1098 are able to abolish the deubiquitinase activity of USP7 (Rouge et al., 2016).

## USP7: One Protein, Multiple Roles

Many proteins have been identified as potential substrates and binding partners of USP7, such as viral proteins, transcription factors, and epigenetic modulators (Figure 8), and most of these substrates play important roles in viral replication, immune response, tumor suppression, epigenetic control, and DNA repair. Here, functions of USP7 on these substrate are as detailed below (Table 1).

**TABLE 1:** Proteins regulated by USP7.

USP7 substrates	Processes	Related cancer	References
EBNA1	Viral proteins		Holowaty et al.,2003
ICP0			Pfoh et al.,2015
vIRFs			Chavoshi et al., 2016; Xiang et al.,2018
LANA			Jager et al.,2012
E1B-55K			Ching et al.,2013
Tat			Ali et al.,2017
Foxp3	Immune response	Non-small cell lung cancer	van Loosdregt et al., 2013; Wang L. et al.,2016
TRIM27		Cervical carcinoma	Cai et al.,2018
NLRP3		Leukemia	Palazon-Riquelme et al.,2018
C-Myc and N-Myc	Oncoproteins	Neuroblastoma	Bhattacharya and Ghosh,2015
p53	Tumor suppressor proteins	Ovarian cancers	Oliner et al., 1992; Li et al.,2002
DAXX		Breast cancer	Tang et al.,2006
PTEN		Chronic lymphocytic leukemia	Morotti et al.,2014
FOXOs family		Lung carcinoma	Huang et al.,2005
DNMT1	Epigenetics	Colon cancer	Du et al., 2010; Bronner,2011
SUMO			Lecona et al.,2016
LSD1		Medulloblastoma	Yi et al.,2016
CHK1	DNA damage and repair		Alonso-de Vega et al.,2014
UVSSA			Sarasin, 2012; Zhang et al.,2012
ANXA1		Hela	Park et al.,2015
XPC			He et al.,2014
HLTF, Rad18			Qing et al.,2011
Polη			Qian et al.,2015
RNF168		Breast cancer	Malapelle et al.,2017
PHF8		Breast cancer	Wang Q. et al.,2016
MDC1		Cervical cancer	Su et al.,2018
Wnt/β-catenin signaling pathway	Several canonical signaling pathways	Colorectal cancer	Novellasdemunt et al.,2017
NF-κB signaling pathway		Multiple myeloma	Colleran et al.,2013
NOTCH signaling pathway		Lymphoblastic leukemia	Shan et al.,2018

### Viral Proteins

#### EBNA1

EBNA1 of EBV is important for the replication, segregation, and transcriptional activation of latent EBV genomes, it has been implicated in host cell immortalization, and avoids proteasome processing and cell-surface presentation. The amino acids 395–450 of EBNA1 bind to the USP7 N-terminal domain with a dissociation constant of 0.9–2 μM. The △395–450 mutant that selectively disrupted the binding to USP7 was found to increase fourfold EBNA1 replication activity than wild-type, but performed no impact on EBNA1 turnover and cell-surface presentation (Holowaty et al., 2003). As p53 and EBNA1 share similar binding sites with USP7, EBNA1 peptide efficiently competes with p53 peptide for USP7 binding, which results the decreasing stability of p53, and protects cells from apoptosis (Saridakis et al., 2005).

#### ICP0

Infected cell protein 0 (ICP0) of HSV is a multifunctional protein containing 775 amino acids that acts as a promiscuous trans-activator linked to the degradation of several proteins. The ^618^PRKCARKT^625^ of ICP0 binds to a negatively charged region on Ubl2, where the residues K620 and K624 of ICP0 form direct contacts with residues D762 and D764 in Ubl2 of USP7 (Pfoh et al., 2015). Overexpression of USP7 had no effect on the mRNA level of ICP0, but could accelerate the mRNA accumulation of thymidine kinase (TK) and gI, which are important for HSV infection of non-replicating cells. The mutations at residues 620 to 626 of ICP0 (named as R6702) can abolish the interaction between USP7 and ICP0, and the replication of R6702 in cells cannot be impaired (Kalamvoki et al., 2012). Hence, inhibition of USP7 and/or its interaction with ICP0 using small molecule inhibitors may decrease the virulence of HSV.

#### vIRFs

Among the vIRFs, vIRF1 could interact with the TRAF domain of USP7 via EGPS motif. The vIRF1 interaction with USP7 can decrease p53 levels by blocking the deubiquitination and stabilization of USP7 on p53. Thus the KSHV could have a lifelong infection when p53 is destabilized by USP7 coupled with vIRF1 (Chavoshi et al., 2016). Besides, vIRF3 is expressed in human herpes virus 8 (HHV-8) – infected primary effusion lymphoma (PEL) cells. The vIRF3 has two copies of EGPS, and both support the vIRF3 – USP7 interaction. This interaction plays important roles in PEL cell growth and viability and contributes to the suppression of productive virus replication (Xiang et al., 2018). For another vIRF family member, amino residues 210–216 of KSHV vIRF4 bind to the same surface groove of the USP7 TRAF domain as that can be recognized by MDM2 and p53. Moreover, the amino residues 202–208 of vIRF4 interact with the β-sheet in TRAF domain. The vIRF4-derived vif1 and vif2 peptides can restore p53 dependent apoptosis in wild-type p53 cancer cells by suppressing the USP7 activity. Thus the two peptides may be considered as potential backbones for peptide mimic small molecule inhibitors development for anti-cancer therapies (Lee et al., 2011).

#### LANA

The viral latency-associated nuclear antigen 1 (LANA) is expressed in all latency KSHV-infected cells and involves in viral latent replication and maintenance of the viral genome. The amino residues 971–986 of LANA interact with TRAF domain of USP7 with similar binding sites as EBNA1, while the △971–986 mutant shows an enhanced ability to replicate latent viral DNA. These results indicate that USP7 may influence accessibility of the viral DNA for latent replication or LANA-mediated viral persistence (Jager et al., 2012). Because of the role of USP7 in EBNA1 – dependent latent replication of EBV, USP7 may play the same role in the replication of latent viral DNA among gamma-1 and gamma-2 herpesviridae.

#### E1B-55K

Adenovirus E1B protein refers to one or two proteins transcribed from the E1B gene of the adenovirus: a 55 kDa protein and a 19 kDa protein. The N-terminal 79 amino acids of E1B-55K interact with the TRAF domain of USP7. Abrogation of USP7 decreases the protein level of E1B-55K and reduces progeny viral production. Therefore, the small inhibitors of USP7 may be used to treat adenovirus infections (Ching et al., 2013).

#### Tat

Human immunodeficiency virus (HIV) Tat is synthesized early after infection and mainly responsible for enhancing viral production. USP7 deubiquitinates and stabilizes Tat and enhances HIV-1 production. In turn, HIV-1 infection leads to the overexpression of USP7. These results show that the small inhibitors of USP7 can be used as a novel anti-HIV approach (Ali et al., 2017).

In sum, these results show that USP7 is recruited by these viruses to promote their survival in the host. So we speculate that USP7 may be an attractive target for controlling infection and other malignancies caused by these viruses.

### Immune Response

#### Foxp3

Recent years, more and more reports have identified the importance of USP7 on keeping T regulatory cells (Treg) functions. As the major factor that restrains autoimmune responses, Treg cell expresses the forkhead transcription factor Foxp3, which is necessary for Treg cell development (Bettelli et al., 2005; Laurence et al., 2013). According to a report in 2016, five distinct lysine residues (K249, K251, K263, K267, and K393) in Foxp3 were identified to be ubiquitinated, and Foxp3 can be stabilized by USP7 mediated deubiquitination, resulting in the maintenance of Treg cell number and function (Wang L. et al., 2016). In fact, a study in 2013 showed that aberrant USP7 overexpression decreases Foxp3 polyubiquitination and protects it from proteasome degradation, resulting in Treg-cell-mediated suppression and tumor growth. On the contrary, USP7 knockdown decreases Foxp3 level and abrogates Treg cell-induced suppression of autoimmune responses *in vitro* and *in vivo* (van Loosdregt et al., 2013).

**FIGURE 3:**
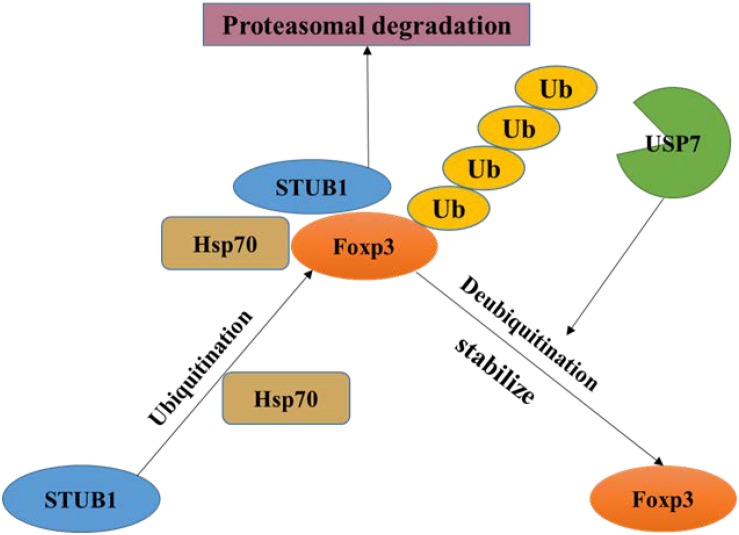
Regulation of Foxp3 by USP7. Foxp3 is ubiquitinated by STUB1 and then produces a complex containing Foxp3, Hsp70 and STUB1, which leading to proteasome degradation of Foxp3. USP7 can remove the ubiquitin on Foxp3 and stabilize it.

Later studies gave the mechanism how the level of Foxp3 is regulated. Foxp3 could be ubiquitinated and degraded by the E3 ubiquitin ligase stress inducible protein 1 homology and U-Box containing protein 1 (STUB1). In addition, Foxp3, Heat Shock Protein 70 (Hsp70) and STUB1 associate together as a complex, indicating that these proteins bind and promote Foxp3 ubiquitination (Figure 3) (van Loosdregt and Coffer, 2014). Moreover, it is found that mesenchymal stem cells (MSCs) – induced Treg cells express high level of USP7 and low level of STUB1. Besides, Foxp3 mRNA expression was positively associated with USP7 and negatively associated with STUB1 (Khosravi et al., 2018). So, it provides us an opportunity to find a new way to study the unique role of USP7 in Treg cells and makes USP7 as a target in immunology.

#### TRIM27

Among the binding partners of USP7, tripartite motif 27 (TRIM27) is an ubiquitin E3 ligase that negatively regulates antiviral signaling by promoting the ubiquitination and degradation of TRAF family member-associated NF-κ-B activator – binding kinase 1 (TBK1). USP7 interacts with TRIM27 and forms the USP7-TRIM27-TBK1 complex, and the interaction between USP7 and TRIM27 can be enhanced after Sendai virus (SeV) infection. When USP7 was overexpressed, TRIM27 can be protected from degradation, which contributed to the ubiquitination and degradation of TBK1, resulting in decreased type I interferons (IFNs) signaling (Cai et al., 2018). As IFNs are a series of signaling proteins which are produced and released by host cells to cope with the presence of pathogens, USP7 can enhance the effects of TRIM27 on TBK1-induced IFN – stimulated response element (ISRE) and IFN-β activation (Zaman et al., 2013). Therefore, USP7 may act as a significant host protein to bridge the viral proteins with the antiviral immune response. Therapeutic methods against the USP7-TRIM27 complex may overcome the immune escape mediated by various viruses.

#### NLRP3

USP7 may also impact on regulating NLR family pyrin domain containing 3 (NLRP3) inflammasome activation. NLRP3 is expressed primarily in macrophages as a component of the inflammasome to monitor products of damaged cells such as extracellular ATP and crystalline uric acid. The ubiquitination status of NLRP3 itself can be altered by USP7 and USP47. Furthermore, researchers discovered that the activity of USP7 and USP47 were augmented once the inflammasome was activated. In the meantime, they discovered that abrogation of both USP7 and USP47 resulted in reduction of inflammasome activation (Palazon-Riquelme et al., 2018).

To sum up, there is a remarkable connection between USP7 and immune-associated proteins, and so many studies have shown that the important roles of USP7 on regulating these proteins. It’s worth thinking about USP7 inhibitors in combination with immunotherapy will be applied to cancer therapy so that the antitumor effect can be promoted. We hope to see their potential dual antitumor activity will be applied to clinical trials on day.

### Oncoproteins

#### C-Myc and N-Myc

There are three members in Myc family: C-Myc, l-Myc, and N-Myc. Myc family is the most frequent amplified oncogene in human, which contributing to the formation of cancer. Among them, C-Myc and N-Myc are the substrates of USP7. USP7 overexpression can promote C-Myc stability by deubiquitination as well as transformation/transcription domain-associated protein (TRRAP), which is an adaptor protein known as a regulator of C-Myc. On the other hand, C-Myc mRNA can be accumulated by TRRAP indirectly (Bhattacharya and Ghosh, 2015).

N-Myc is another transcription factor that can be stabilized by USP7 via deubiquitination (Tavana et al., 2016). Hence, USP7 inhibitor p5091 was applied to decrease N-Myc expression in a dose dependent manner in neuroblastoma (Tavana et al., 2016). As a consequence, USP7 can be considered as a drug target to modulate C-Myc and N-Myc amount in order to block tumor development.

### Tumor Suppressor Proteins

#### p53

**FIGURE 4:**
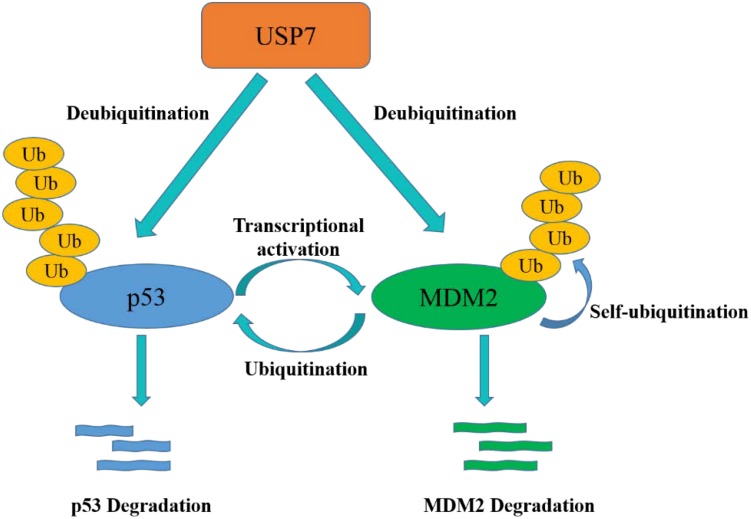
USP7-p53-MDM2 axis interactions control the stability of p53 and MDM2. USP7 can stabilize p53 by deubiquitination, meanwhile, USP7 can also remove the ubiquitin of MDM2, which promotes to p53 degradation.

p53 participates in cell cycle arrest, DNA repair, apoptosis, senescence and plays a key role in maintaining normal cell growth (Levine, 1997). USP7 plays a paradoxical role in regulating p53 functions through a variety of mechanisms. On one hand, p53 binds to TRAF domain and C-terminal (amino acids 880–1050) of USP7, and then USP7 ubiquitinates p53 directly and prevents it from degradation. On the other hand, TRAF domain and C-terminal (amino acids 801–1050) of USP7 can interact with MDM2 to increase its stability by erasing the ubiquitin on MDM2, an E3 ligase of p53 (Oliner et al., 1992), and protect it from proteasome degradation. Subsequently, MDM2 ubiquitinates p53 and causes its proteasome degradation, resulting in low expression of p53 in cancer cells (Figure 4) (Li et al., 2002, 2004). In addition, MDM2 can also inhibit the transcription of p53 (Wade et al., 2013). Therefore, inhibition of the interaction between MDM2 and p53 can stabilize p53 (Vassilev et al., 2004). It is noteworthy that, crystal structures analysis and binding studies suggest that the MDM2 peptide and p53 peptide bind to the same surface groove in USP7, but MDM2 performs more extensive interaction and stronger affinity (Hu et al., 2006). Taken together, the activation of USP7-MDM2-p53 interaction can promote the occurrence and development of tumors. The design of small molecules that disrupt or prevent the interaction may be an important target for cancer therapy by regulating p53 pathway.

#### DAXX

Death-domain-associated protein (DAXX) is a highly conserved and developmentally essential nuclear protein, which participates in many cellular processes (Lindsay et al., 2008). The N-terminal 160 amino acids and amino acids 347–570 of DAXX associate with USP7, which are far from the binding sites of MDM2 on DAXX. In unstressed cells, DAXX interacts with USP7 and MDM2, and mediates the stabilization of USP7 on MDM2, thus blocking p53 activation. In response to DNA damage, self-ubiquitination of MDM2 is accelerated when MDM2 is stripped from DAXX and USP7. That is to say, DAXX directs the ligase activity of MDM2 through regulating USP7 (Tang et al., 2006). Recent reports also show that USP7 and DAXX are critical in regulating the correct execution of mitosis by forming a tertiary complex as MDM2/DAXX/USP7 (Zhang et al., 2010). DAXX binding increases USP7 activity toward MDM2. Disassemble the MDM2-DAXX-USP7 complex can increase MDM2 self-ubiquitination and degradation, which leads to the stabilization and accumulation of p53 (Kumar et al., 2018).

#### PTEN

Phosphatase and tensin homolog (PTEN) is a tumor suppressor gene that displays dual specific phosphatase activity. PTEN inhibits the proliferation and migration of tumor cells (Blanco-Aparicio et al., 2007). It is reported that nuclear PTEN import is promoted by its mono-ubiquitination (Trotman et al., 2007). However, USP7 can remove the mono-ubiquitination of PTEN, triggering its nuclear exclusion and PTEN inactivation (Morotti et al., 2014). Likewise, USP7 inhibitor, P5091, regains PTEN nuclear pool and restores its tumor suppressive functions in chronic lymphocytic leukemia (CLL) (Carra et al., 2017). In addition, PTEN deletion leads to accumulation of activated AKT, and subsequent phosphorylation of MDM2 by AKT (Blanco-Aparicio et al., 2007), which results in the ubiquitination and degradation of p53 (Freeman et al., 2003). Therefore, PTEN deficiency causes p53-dependent cancer-promoting processes. This suggests how important it is to inhibit USP7 to ensure PTEN protein localization and activity.

#### FOXOs Family

The Forkhead box O (FOXO) family members, including FOXO1, FOXO3, FOXO4 and FOXO6, are transcription factors that take part in regulating several cellular responses, including cell cycle progression and apoptosis and so on (van der Horst and Burgering, 2007). It is reported that USP7 can remove ubiquitin from FOXO1, which is written by Skp2 as an E3 ligase (Huang et al., 2005). Besides, mono-ubiquitination FOXO4 localizes in the nucleus and exhibits stronger transcriptional promotion activity (Brenkman et al., 2008). USP7 can suppress FOXO4 activity due to its deubiquitination and re-localization (van der Horst et al., 2006). In a word, USP7 affects tumor progression by interacting with FOXOs and affecting their activity and localization.

### Epigenetics

#### DNMT1

**FIGURE 5:**
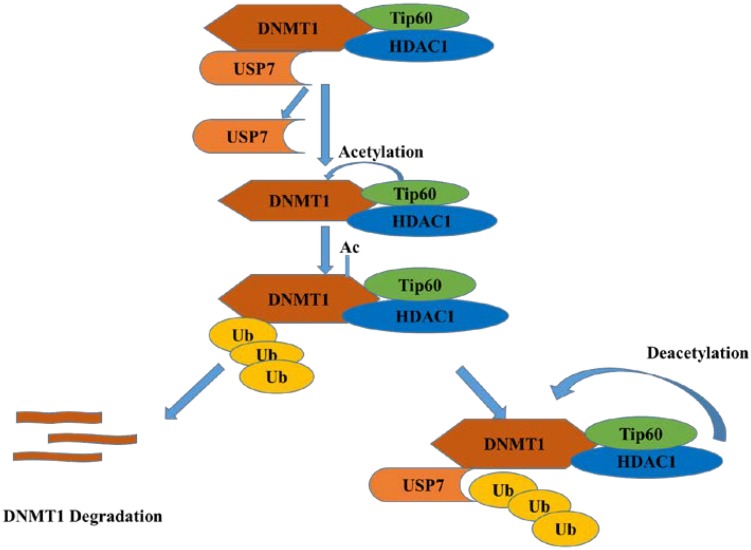
USP7 and HDAC1 protect DNMT1 from degradation while Tip60 acetylates DNMT1 and promotes its degradation.

DNMT1 (DNA methyltransferase 1) contributes to the maintenance of DNA methylation. As reported, USP7 can deubiquitinate and stabilize DNMT1 when its acetylation is erased by histone deacetylase 1 (HDAC1), which protects DNMT1 from proteasome degradation (Bronner, 2011). When the KG linker of DNMT1 is acetylated by Tip60, USP7 breaks away from DNMT1 and results in the degradation of DNMT1 mediated by proteasome system (Figure 5) (Du et al., 2010). Thus, HDAC and USP7 inhibitors can be applied in combination for cancer treatment (Cheng et al., 2015).

#### SUMO

Small ubiquitin related modifier (SUMO) is a ubiquitin-like molecule, which binds to its substrate by E3 SUMO ligase in a similar way as ubiquitination (Geoffroy and Hay, 2009). Like ubiquitin, proteins can be SUMOylated as mono-SUMOylation or poly-SUMOylation, but differently, poly-SUMOylation cannot lead to target degradation directly (Smits and Freire, 2016). Recent research shows that USP7 is associated with DNA synthesis (Smits and Freire, 2016). USP7 associates with an active DNA replication fork and inhibition of USP7 can reduce DNA replication. Moreover, Lecona et al. (2016) identified SUMO2 as a new USP7 substrate and demonstrated that USP7 can deubiquitinate SUMO2 *in vitro* and *in vivo*. However, the fate of SUMO2 after deubiquitination and its biological function are still unclear (Lecona et al., 2016).

#### LSD1

Histone lysine specific demethylase 1 (LSD1) is the first histone demethylase identified in 2004 and can remove methyl groups of histone H3K4, H3K9 (Shi et al., 2004). As reported, LSD1 can be ubiquitinated by E3 ligase JADE2 (Han et al., 2014). Since ubiquitination of LSD1 is considered as reversible process as ubiquitination and deubiquitination always exit in pair, LSD1 was identified to be deubiquitinated by USP7 and protected it from proteasome degradation (Yi et al., 2016). Besides, patients with high expression of USP7, REST, and LSD1 performed poorer outcomes in medulloblastoma (Callegari et al., 2018). And they found that p53 was a vital downstream transcription factor in the action of USP7 and LSD1.

### DNA Damage and Repair

#### CHK1

USP7 can regulate CHK1 in three manners. The first one is the indirect regulation, USP7 deubiquitinated and prolonged the half-life of Claspin, which leaded to the sustaining phosphorylation of checkpoint kinase 1 (Chk1) in response to genotoxic stress (Faustrup et al., 2009). For the rest two manners, in DNA damage, USP7 deubiquitinates and stabilizes Chk1 via direct deubiquitination in the presence of zinc finger E-box binding homeobox 1 (ZEB1) (Zhang et al., 2014) or not (Alonso-de Vega et al., 2014), while ZEB1 binds to USP7 may result in promoting homologous recombinant-dependent DNA repair and resistant to radiation. In addition, USP7 can also directly regulate the stability of CDC25A, a Cdk-activating phosphatase as the substrate of CHK1, with the aid of brain and reproductive organ expressed protein (BRE). These results show that USP7 is an important modulator of Chk1.

#### CHFR

**FIGURE 6:**
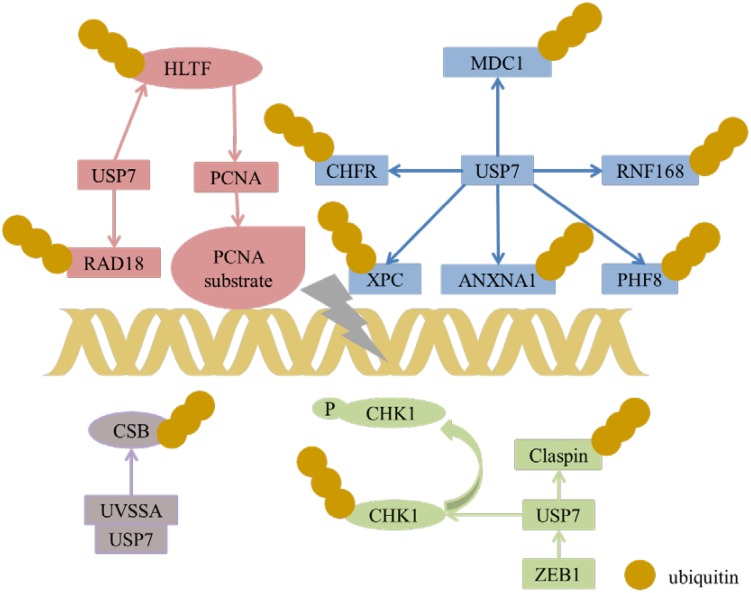
USP7 interacts with a number of substrates in DNA damage response.

Checkpoint with Forkhead and Ring domains (CHFR), a RING family Ub-ligase, is a mitotic checkpoint that delays the transition to metaphase in response to mitotic stress. USP7 binds with CHFR *in vivo* and regulates its stability (Figure 6). These results indicate that USP7 may play a role in the cell cycle progression via the deubiquitination of CHFR (Oh et al., 2007).

#### UVSSA

Transcription-coupled nucleotide excision repair (TC-NER) removes DNA damage of actively transcribed genes. Defect in TC-NER is associated with cockayne syndrome (CS) and ultraviolet – sensitive syndrome (UVSS). Cockayne syndrome B (CSB/ERCC6) and UVSS protein are two important proteins in TC-NER. UVSSA binds with USP7 to stabilize CSB and restores the hypophosphorylated form of RNA polymerase II after UV irradiation (Figure 6) (Zhang et al., 2012). UVSSA and USP7 play roles in controlling the fate of stalled RNA polymerase II, the steady-state level of CSB, the efficiency of TC-NER and cell survival following DNA damage (Sarasin, 2012).

#### ANXA1

ANXA1 is a 37-kDa protein identified as the first member of the annexin superfamily. In response to DNA damage, ANXA1 is cleaved and generates the N-terminal fragment (Ac2-26) and the cleaved form of ANXA1. Both the full length of ANXA1 and Ac2-26 can be translocated to the cell membrane and induce apoptotic cell clearance through recruiting monocytes. The N-terminal of ANXA1 shares the USP7-binding motif sequences (AMVS and ALLS) and interacts with USP7. Hence, USP7 can deubiquitinate and stabilize ANXA1 (Figure 6). USP7 may participate in the DDR after UV-induced DNA damage in certain types via ANXA1 (Park et al., 2015).

#### XPC

Xeroderma pigmentosum complementation group C (XPC) is a critical damage recognition factor which binds to helix-distorting DNA lesions and initiates nucleotide excision repair (NER). During the early stage of NER of UV light-induced DNA lesions, XPC is ubiquitinated. Ubl1 domain (amino acids 560–644) of USP7 can bind and erase the ubiquitination on XPC and prevents XPC from proteolysis (Figure 6). Taken together, USP7 plays a vital role in regulating NER through deubiqitinating XPC (He et al., 2014).

#### HLTF, Rad18, and Polη

Helicase-like transcription factor (HLTF) is a double-stranded DNA translocase that can promote the polyubiquitination of proliferating cell nuclear antigen (PCNA), while Rad6–Rad18 monoubiquitinates PCNA, both of which make PCNA work as a molecular switch between various DNA damage bypass processes. On one hand, USP7 stabilizes HLTF after genotoxic stress, resulting in prolonging the half-life of HLTF, thus in turn increases polyubiquitination of PCNA (Figure 6) (Qing et al., 2011). Besides, USP7 and DNA polymerase eta (Polη), a key player in several DNA damage-tolerance pathways, interact with each other, and USP7 increases UV-induced PCNA ubiquitination through stabilizing Polη and in turn facilitates the recruitment of DNA translesion synthesis (TLS) polymerases to bypass DNA lesions. Therefore USP7 promotes monoUb-PCNA mediated stress-tolerance pathways via the stabilization of Polη. These results provide new mechanistic for USP7-related tumorigenesis and therapeutic strategy (Qian et al., 2015). On the other hand, the amino acids 110–251 of Rad18 interact with USP7 and contain two USP7-binding motifs. Loss of USP7 destabilizes Rad18 and compromises UV-induced PCNA monoubiquitylation and Polη recruitment to stalled replication forks (Zlatanou et al., 2016).

#### RNF168

During DDR, histone ubiquitination by RNF168 orchestrates the recruitment of downstream DDR factors, e.g., breast cancer type 1 susceptibility protein (BRCA1) and p53 binding protein 1 (53BP1). The Ubl1 domain of USP7 binds to RNF168 (Figure 6). USP7 regulates H2A monoubiquitination and H2A/X polyubiquitination via its regulation on RNF168. In summary, USP7 plays a vital role in regulation of Ub-dependent signaling in DDR via monitoring RNF168 (Malapelle et al., 2017).

#### PHF8

Plant homeodomain finger-containing protein 8 (PHF8) consists of an N-terminal plant homeodomain and recognizes and binds tri-methyl histone 3 lysine 4 at transcription start sites. The C-terminal region of PHF8 binds with the TRAF domain of USP7, and USP7 promotes the stability of PHF8 via deubiquitinase activity and contributes to the maintenance of genome integrity, which is implemented in DDR (Figure 6). The USP7/PHF8 is involved in breast carcinogenesis, indicating these molecules may be as potential targets for breast cancer intervention (Wang Q. et al., 2016).

#### MDC1

DNA damage checkpoint protein 1 (MDC1) is important for the initiation and amplification of the DDR. USP7 deubiquitinates and stabilizes of MDC1, resulting in sustaining the DDR, while depletion of USP7 influences the engagement of MRE11-RAD50-NBS1(MRN)-MDC1 complex and the recruitment of the downstream factors 53BP1 and BRCA1 at DNA lesions. USP7 promotes cervical cancer cell survival and confrere cellular resistance to genotoxic insults via the stabilization of MDC1 (Su et al., 2018).

In a nutshell, USP7 plays a vital role in the DNA damage response (Figure 6), and it can be targeted for the treatment of malignancies with DDR defects. Besides, USP7 inhibitors can be combined with genotoxic agents as a novel therapeutic strategy for the treatment of cancer.

USP7 stabilizes HLTF to result in polyubiquitination of PCNA and induces monoubiquitination of PCNA through regulating Rad18. USP7 plays a role in DDR through regulating MDC1, CHFR, XPC, ANXNA1, RNF168, and PHF8. USP7 and UVSSA interact with each other to control steady state of CSB following DNA damage. USP7 can regulate stability of CDC25A via deubiquitination CDC25A directly and through regulating Claspin and CHK1 expression.

### Several Canonical Signaling Pathways

#### Wnt/β-Catenin Signaling Pathway

**FIGURE 7:**
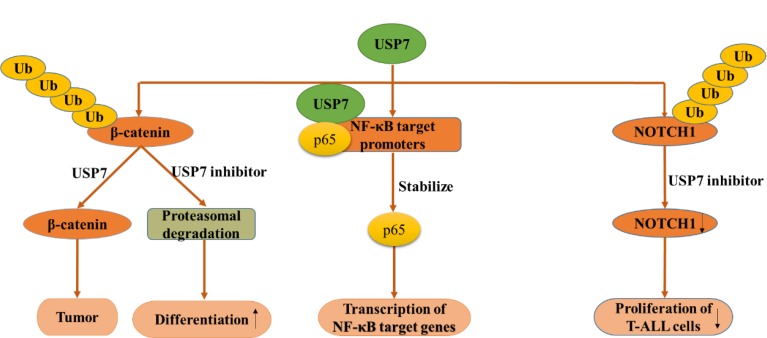
USP7 functions in several canonical signaling pathways. USP7 functions in Wnt/β-catenin signaling pathway **(left)**, NF-κB signaling pathway **(middle)**, NOTCH signaling pathway **(right)**.

Wnt signaling was initially found for its function in cancer and embryonic development and then was found responsible for tissue regeneration in adult bone marrow, skin and intestine. β-Catenin, a key element in Wnt signaling pathway, is regulated by diverse PTMs, including ubiquitination (Ma et al., 2014). According to a research in 2017, β-catenin can be deubiquitinated and stabilized by USP7 in adenomatous polyposis coli (APC) truncating mutated colorectal cancer (CRC) but not APC wide type CRC, which resulting in the activation of Wnt pathway (Novellasdemunt et al., 2017). Mechanism study suggested that APC β-catenin inhibitory domain (CID) protects β-catenin from USP7-mediated deubiquitination, while APC lacking CID exposes β-catenin to USP7 for deubiquitination. Hence, abrogation of USP7 in APC-mutated CRC suppresses Wnt activation by regaining β-catenin ubiquitination, which leads to the cell differentiation, and inhibits tumor growth (Novellasdemunt et al., 2017). With the aid of USP7 inhibitor P5091, Wnt pathway can be inactivated by improving ubiquitination and degradation of β-catenin, which provides evidence for the rationality for developing USP7 inhibitors as anti-CRC agent (Figure 7A) (An et al., 2017). In a nutshell, USP7 can be considered as a Wnt activator for tumor-specific therapeutic target for most CRCs.

**FIGURE 8:**
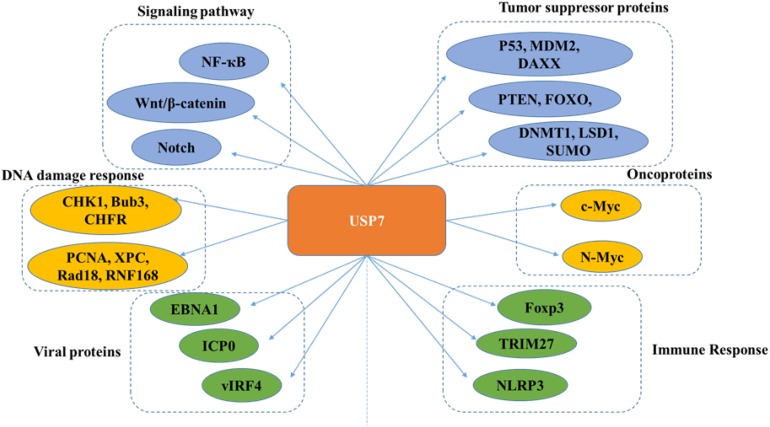
Overview of USP7 functions.

#### NF-κB Signaling Pathway

Nuclear factor kappa B (NF-κB) signaling pathway is responsible for the transcription of a series of genes that controlling inflammation and immunity. As an essential regulator of Toll-like-receptor (TLR) and tumor necrosis factor receptor (TNFR)-inducible inflammatory gene expression, NF-κB is regulated by USP7 in a research in 2013. Different from other USP7 partners and substrates, NF-κB p65 and USP7 interact together after USP7 is recruited to NF-κB target promoters. Besides, the inhibition of USP7 lead to decreased TLR and TNFR-induced expression of Interleukin (IL-6), TNFα (NF-κB reporter) indicates that the deubiquitination of NF-κB by USP7 may have therapeutic potential (Figure 7B) (Colleran et al., 2013).

In 2018, some researchers found that knockout of USP7 dramatically increased the sensitivity of multiple myeloma (MM) cells to bortezomib (BTZ) which led to myeloma cell death and inhibited NF-κB activation by stabilizing IκBα. As expected, usage of USP7 inhibitors also inhibited the activation of NF-κB and the combination of USP7 inhibitor with BTZ triggered the synergistic antitumor activity in bortezomib-resistant MM cells. Taken together, this study provides a new application for USP7 inhibitors alone or in combination with BTZ to overcome BTZ resistance and improve the patient prognosis in MM (Yao et al., 2018).

In all, several reports have illustrated the mechanism that how USP7 and its related proteins regulate NF-κB signaling pathway. However, more deep studies should be conducted to make the mechanism more clearly and logically and there are still great challenges for researchers to face.

#### NOTCH Signaling Pathway

Notch signaling pathway is highly conserved and presents in most multicellular organisms. This intercellular signaling cascade is involved in cell differentiation, proliferation, and contributes to the fate of cells and occurs in multiple organisms and tissues, containing early T cell development in the thymus and peripheral T cell differentiation (Auderset et al., 2012; Bailis et al., 2013; Amsen et al., 2015). There are four notch receptors in mammals possessing NOTCH1-4 in which NOTCH1 can be stabilized through USP7-mediated deubiquitination. Previous studies have revealed that ubiquitination regulates the stability, activity, and localization of NOTCH1. However, the specific deubiquitinase that affects NOTCH1 protein stability was clarified recently. Researchers reported that USP7 can deubiquitinate and stabilize NOTCH1 *in vivo* and *in vitro*, on the other hand, knockdown of USP7 increased the ubiquitination of NOTCH1. Used up of USP7 significantly restrained the proliferation of T-cell acute lymphoblastic leukemia (T-ALL) cells *in vitro* and *in vivo*, accompanied by downregulation of the NOTCH1 protein level, suggesting that targeting the USP7/NOTCH1 axis is a novel strategy to combat T-ALL and other NOTCH1-related malignancies (Figure 7C) (Shan et al., 2018). Almost at the same time, researchers found USP7 can bind several oncogenes by interacting and stabilizing NOTCH1 and JmjC Domain-Containing Protein 3 (JMJD3) in order to control leukemia growth. What’s more, USP7 and NOTCH1 bind T-ALL superenhancers, and inhibition of USP7 leads to a decrease of the transcriptional levels of NOTCH1 targets and T-ALL cell growth *in vitro* and *in vivo*. Therefore, USP7 cooperating with NOTCH1 can improve the oncogenic transcriptional program in T-ALL (Jin et al., 2018).

The functions of USP7 on different signaling pathways indicate the brand new role of USP7 as a great target. To be sure, other classical signaling pathways which may be regulated by USP7 is yet to be found. It provides us a great challenge to find the new mechanisms between USP7 and other classical signaling pathways.

### USP7 in Cancer

USP7 is highly expressed in a wide variety of cancers and affects the progression of cancer diseases. Moreover, USP7 assumes different roles in different tumors. In prostate cancer, high expression of USP7 is directly related to tumor invasion (Song et al., 2008). USP7 plays a key role in carcinogenesis via p53-dependent pathways in non-small cell lung carcinoma (NSCLCs) (Masuya et al., 2006). Studies have shown that changes of USP7 regulate colon carcinoma growth and apoptotic sensitivity *in vivo* (Becker et al., 2008). USP7 maintains DNA damage response and promotes cervical cancer, and is positively correlated with poor survival rate in patients with cervical cancer (Su et al., 2018). USP7 regulates human terminal erythroid differentiation by stabilizing GATA1, providing a certain treatment for leukemia (Liang et al., 2019). In short, USP7 plays an important role in a variety of pathologies and is a good target from a therapeutic point of view.

## USP7 Inhibitors

**FIGURE 9:**
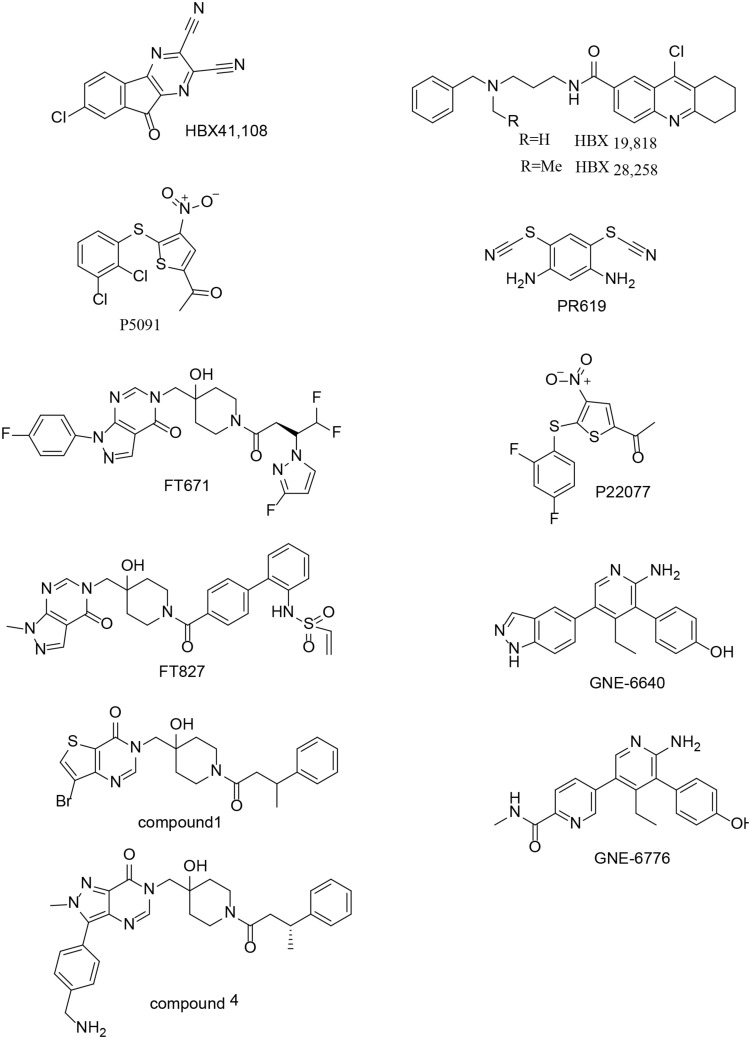
Chemical structures of USP7 inhibitors.

**FIGURE 10:**
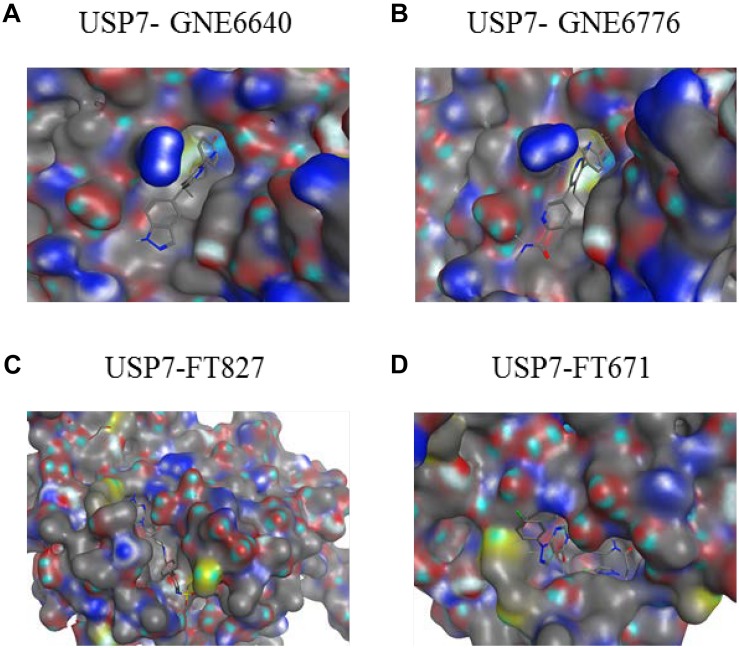
Co-crystal structures of USP7 in complex with inhibitors. The electrostatic surface representation of the CD of USP7 is shown along with compounds **(A)** USP7-GNE6640 (PDB code 5UQV). **(B)** USP7-GNE6776 (PDB code 5UQX). **(C)** USP7-FT827 (PDB code 5NGF). **(D)** USP7-FT671 (PDB code 5NGE). The images were generated with molecular operating environment (MOE).

USP7 is a promising target not only for its roles in cellular pathways including regulators of viral proteins, immune response, oncogenes, and DNA damage but also because of its aberrant expression in various cancers. Due to lack of co-crystal structures between USP7 and small molecule inhibitors, there is no potent and selective USP7 inhibitor for a long time (Colland et al., 2009; Altun et al., 2011; Chauhan et al., 2012; Reverdy et al., 2012) (Figure 9). However, several groups reported the structures of USP7 in complex with small molecule inhibitors last year (Kategaya et al., 2017; Turnbull et al., 2017) (Figure 10) and these structures gives guidance to obtain structure-based small molecule inhibitors.

## Conclusion and Future Perspectives

This review illustrates our current knowledge of USP7, including its source and characterization, structure, binding partners and substrates in various biological processes. Besides, how USP7 regulates various aspects of a cell under both normal and pathological states are elaborated in detail. As the processes of ubiquitination and deubiquitination are extremely dynamic and context-specific, a series of studies have linked USP7 to different cancers. The biology, particularly the immune oncology mechanisms, reveal that USP7 inhibitors would be useful drugs, thus it is vital to develop highly selective and specific inhibitors of USP7. The association of USP7 with several canonical signaling pathways still needs characterized in order to search new targets and regulatory mechanisms. Last but not least, USP7 may be a promising target for cancer therapy and it therefore merits further studies.

## Author Contributions

ZW discusses the structure of USP7, the viral protein targets of USP7, the DNA damage substrates of USP7 and conclusion. WK talks about the introduction, immune function of USP7, Wnt/β-catenin signaling pathway, and NF-κB signaling pathway. YY illustrates oncoproteins and tumor suppressor proteins. JP states about the epigenetics substrates of USP7. HR talks about the NOTCH signaling pathway. ZS and YZ provide expertise and feedback. HL provides funding.

## Conflict of Interest Statement

The authors declare that the research was conducted in the absence of any commercial or financial relationships that could be construed as a potential conflict of interest.
